# Is the Power Spectrum of Electromyography Signal a Feasible Tool to Estimate Muscle Fiber Composition in Patients with COPD?

**DOI:** 10.3390/jcm10173815

**Published:** 2021-08-25

**Authors:** Antonino Casabona, Maria Stella Valle, Luca Laudani, Claudia Crimi, Cristina Russo, Lucia Malaguarnera, Nunzio Crimi, Matteo Cioni

**Affiliations:** 1 Laboratory of Neuro-Biomechanics, Department of Biomedical and Biotechnological Sciences, School of Medicine, University of Catania, 95123 Catania, Italy; casabona@unict.it (A.C.); llaudani@cardiffmet.ac.uk (L.L.); mcioni@unict.it (M.C.); 2 Cardiff School of Sport and Health Sciences, Cardiff Metropolitan University, Cardiff CF5 2YB, UK; 3 Respiratory Medicine Unit, Policlinico Vittorio Emanuele-San Marco, University Hospital, 95123 Catania, Italy; dott.claudiacrimi@gmail.com; 4 Section of Pathology, Department of Biomedical and Biotechnological Sciences, University of Catania, 95123 Catania, Italy; cristina.russo87@alice.it (C.R.); lucmal@unict.it (L.M.); 5 Department of Clinical and Experimental Medicine, University of Catania, 95123 Catania, Italy; nunzio.crimi@unict.it; 6 Gait and Posture Analysis Laboratory, Policlinico Vittorio Emanuele-San Marco, University Hospital, 95123 Catania, Italy

**Keywords:** musculoskeletal system, electromyography, kinematics, knee joint, mean frequency, median frequency, regression analysis, principal component analysis

## Abstract

A greater proportion of glycolytic muscle fibers is a manifestation of skeletal muscle dysfunction in Chronic Obstructive Pulmonary Disease (COPD). Here, we propose to use the spectral analysis of the electromyographic signal as a non-invasive approach to investigate the fiber muscle composition in COPD. We recorded the electromyographic activity of Rectus Femoris (RF), Vastus Lateralis (VL), Vastus Medialis (VM) and Biceps Femoris (BF) muscles, in ten patients and ten healthy individuals, during non-fatiguing, flexion–extension leg movements. The mean (MNF) and median frequencies (MDF) were calculated, and the most common profiles of electromyographic power spectrum were characterized by using the principal component analysis. Frequency parameters showed higher values in patients with COPD than in the control group for the RF (+25% for MNF; +21% for MNF), VL (+16% for MNF; 16% for MNF) and VM (+22% for MNF; 22% for MNF) muscles during the extension movements and for the BF (+26% for MNF; 34% for MNF) muscle during flexion movements. Spectrum profiles of the COPD patients shifted towards the higher frequencies, and the changes in frequency parameters were correlated with the level of disease severity. This shift of frequencies may indicate an increase in glycolytic muscle fibers in patients with COPD. These results, along with the non-fatigable nature of the motor task and the adoption of a non-invasive method, encourage to use electromyographic spectral analysis for estimating muscle fiber composition in patients with COPD.

## 1. Introduction

Dysfunction of skeletal muscles is a relevant manifestation of Chronic Obstructive Pulmonary Disease (COPD) that affects motor function and limits the ability to carry out habitual activities of daily life [1,2,3,4,5]. This dysfunction represents an influent comorbidity, both in respiratory and peripheral muscle groups, derived from primary effects of COPD, such as alterations in ventilatory mechanics and oxygen supply limitations [4]. As the severity of COPD increases over time, peripheral skeletal muscles show several biochemical and histological alterations consisting of a reduction in cross-sectional area [6,7], enzymatic and mitochondrial abnormalities [8] and changes in the composition of the muscle fiber types [9,10,11]. These alterations contribute to a progressive decrease in muscle strength and endurance, especially in the lower limb muscles [12,13].

While muscle strength and endurance can be assessed by non-invasive tests, such as incremental isometric contractions or maximized walking performance within a time-interval [4,5], histological and biochemical examinations may require invasive techniques, including biopsy. However, in the case of studying the fiber composition of a muscle, recording the surface electromyography (sEMG) signal could be a non-invasive, valid alternative method [14,15].

A typical application of sEMG is to evaluate time-domain parameters, such as amplitude or latency of electrical muscle activation. Conversely, the analysis of spectral content of the sEMG signal provides frequency-domain parameters by which the muscle fiber composition can be estimated. In fact, functional relationships between fiber type composition and response in spectral content of the sEMG have been documented by comparing changes in frequency bandwidth (i.e., the width of a frequency range) with histochemical measurements of muscle fiber type composition [16] or analyzing changes in the activity of muscles with different fiber composition [17]. In these studies, the muscle with a higher content of fast-glycolytic fibers (type II) showed a shift of the sEMG power spectrum profile toward higher frequencies, while spectrum profiles with much lower frequencies were associated with muscle composition with greater percent of slow-oxidative fibers (type I).

Muscle biopsies revealed that patients with COPD show a high proportion of type II fibers [9,10,11,18], especially those with moderate or severe disease that show a greater propensity to switch from type I to type II fibers [11,18]. A high proportion of fast-glycolytic fibers in patients with COPD would suggest that these patients should exhibit an sEMG power spectrum shifted toward higher frequencies when compared with healthy individuals. This prediction was confirmed by several experimental findings [19,20,21,22], supporting the possibility that change in frequency bandwidth in the sEMG power spectrum is a valid tool to estimate muscle fiber composition in patients with COPD. However, since the main goal of these papers was to adopt frequency-domain features as indices of muscle fatigue, incremental fatiguing motor tasks were engaged. In order to use the sEMG power spectrum to only estimate the characteristics of muscle fibers involved in a muscle contraction, a non-fatiguing movement task is preferable. In fact, the contribution of motor unit recruitment to the increase in muscle strength is limited to low and medium levels of contraction, whereas high levels of muscle strength are mainly sustained by an increase in the rate of neuronal firing, without the recruitment of new units [23]. Moreover, in patients with COPD, the level of impairment of muscle function and physical performance could exacerbate during a fatiguing task, limiting the ability of sEMG analysis to reliably detect the changes in muscle fiber composition [7,20,24,25]. 

To date, there is a lack of literature on easy-to-administer ways of identifying and quantifying the disease-induced changes in sEMG frequency during non-fatiguing movement tasks. In the current study, the sEMG power spectrum was obtained from leg flexor and extensor muscles during simple, non-fatiguing knee flexion–extension movements. To test whether this motor task is feasible to show a shift of the sEMG power spectrum toward high frequencies, the mean frequency (MNF) and median frequency (MDF) were computed. Moreover, a characterization of the shape of spectrum profiles was performed by means of principal component analysis (PCA). Finally, we tested the sensitivity of the measured frequency-domain features with respect to the level of disease severity. 

## 2. Material and Methods

### 2.1. Participants

Ten non-hospitalized patients with moderate to severe COPD, under medical supervision at the Respiratory Medicine Unit, Policlinico Universitario di Catania (Catania, Italy), were recruited for this study. Ten healthy subjects, with anthropometric characteristics matching those of the patients, participated as a control group. In Table 1 are reported the anthropometric data from both groups. No significant differences between groups were detected for the age (*p* = 0.801), height (*p* = 0.851) and weight (*p* = 0.835).

The criteria of inclusion to participate to this study were the following: diagnosis of COPD according to Chronic Obstructive Lung Disease (GOLD) criteria, no disease exacerbation within the four weeks prior to the beginning of the study. The fixed exclusion criteria were presence of heart diseases, musculoskeletal traumas and/or rheumatologic diseases. The diagnosis of COPD was based on instrumental investigations such as chest X-ray and most common lung function tests. The tests measured the following parameters: forced vital capacity (FVC), forced expiratory volume in one second (FEV1), FEV1/FVC ratio, the diffusing capacity for carbon monoxide (D_LCO_) and the degree of the sensation of shortness of breath in relation to the accomplishment of common daily activities estimated by the modified Medical Research Council Questionnaire (mMRC). In Table 2, we reported the listing of these lung function tests, measured for each patient.

### 2.2. Experimental Setup, Procedures and Signal Processing

**Figure 1:**
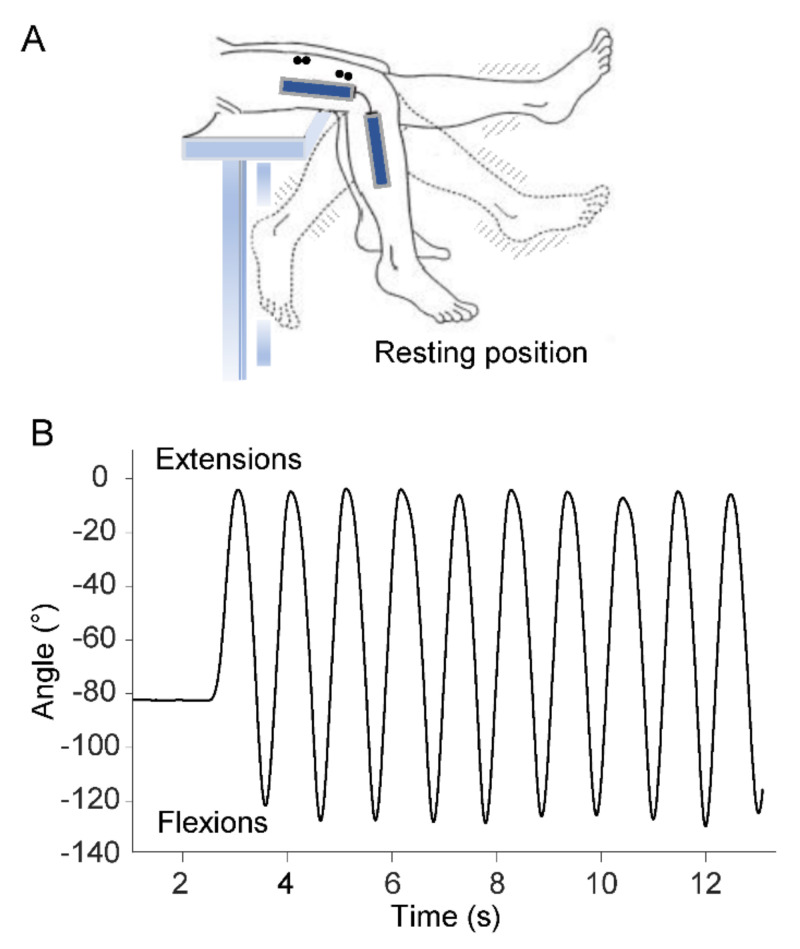
Experimental setup. (**A**) Leg oscillations during voluntary flexion–extension movements. An electrogoniometer acquired the knee joint angle, and four pairs of surface electrodes recorded the sEMG activity from Rectus Femoris, Vastus Lateralis, Vastus Medialis and Biceps Femoris muscles (the last two pairs of electrodes not visible in the figure). (**B**) Representative trajectory of extension and flexion movements.

Participants sat on a medical couch with their knees flexed at resting position and their legs dangling and not touching the ground (Figure 1A). After receiving verbal instructions and demonstration of the procedure, participants were asked to perform a series of ten voluntary flexion–extension movements of the lower limb at maximal range of motion in the sagittal plane (Figure 1B). Angular displacements of the swing movements were measured by means of an electrogoniometer (Biometrics Ltd., Gwent, UK) placed on the lateral surface of the knee joint. Kinematic data were filtered using a low-pass, zero-lag, second-order Butterworth filter with 5 Hz cutoff frequency. 

From the above kinematic measurements, the following timing parameters were computed (Figure 1B): time at movement onset and time at each peak extension and flexion movement.

To analyze muscle activity during the movements, the sEMG signals were recorded by four pairs of electrodes placed on following muscle bellies, according to the SENIAM protocol [26]: Rectus Femoris (RF), Vastus lateralis (VL), Vastus Medialis (VM) and the Biceps Femoris caput longus (BF). The reason of selecting these muscles arose in part from the fact that studies examining histological or electrophysiological characteristics of peripheral muscles in patients with COPD have largely used the VL muscle or other quadriceps components [1,6,7,8,9,10,11,18,19,20,21]. Furthermore, considering that our task consisted of alternating flexion and extension movements, we found it appropriate to expand the sEMG recording to the BF. In fact, this muscle provides an important contribution to the flexion knee movement, and considering the participants’ sitting position, it is more easily accessible than other knee flexors.

The sEMG signal was amplified and high-pass filtered (20 Hz) to remove low-frequency artifacts produced by electrode movements. After full-wave rectification of the raw sEMG signal, the average level of sEMG background recorded 500 ms before the onset of the first extension was subtracted from the sEMG activity acquired during the leg movements. From the rectified signal, a low-pass filter (10 Hz cut-off, 2 pass, 2nd Butterworth) [27] allowed to envelope sEMG activity, and the areas over the ten extensions and flexions were obtained by computing the integral of the sEMG signal. 

Spectral analysis was performed offline on the raw sEMG data segmented in time windows corresponding to every consecutive flexion and extension leg movement. For each of these time intervals, Fast-Fourier transformation was performed using 512 points, and the power spectral density (PSD) of the sEMG signal was obtained. The mean (*MNF*) and median frequencies (*MDF*) were the frequency-domain features computed from the PSD using the following conventional equations:

(1) $$MNF=\overset{M}{\underset{j=1}{\sum }}f_{j}P_{j}/\overset{M}{\underset{j=1}{\sum }}P_{j}$$

(2) $$\overset{MDF}{\underset{j=1}{\sum }}P_{j}=\overset{M}{\underset{j=MDF}{\sum }}P_{j}=\frac{1}{2}\overset{M}{\underset{j=1}{\sum }}P_{j}$$

where *f_j_* is the frequency value of sEMG power spectrum at the frequency bin *j*, *P_j_* is the EMG power spectrum at the frequency bin *j*, and *M* is the length of frequency bin. 

Kinematic and sEMG measurements were recorded and synchronized by using a portable device (POCKET-EMG by Bioengineering Technology and System—BTS, Garbagnate Milanese, Italy). All the signals were sampled at 1000 Hz, with kinematic data resampled at 200 Hz for further processing.

Signal processing was performed by using Matlab version R2020b (Mathworks Inc., Natick, MA, USA).

### 2.3. Statistical Analysis

Preliminary analyses were performed to verify the presence of a normal distribution of the sample (Shapiro–Wilk test) and to verify the homogeneity of the variance between the groups when parametric statistics is applied on a small sample (Levene’s test). 

Appling the Shapiro–Wilk test to check the sEMG data for normality, we found values of *p* greater than 0.05 for both the changes in time-domain sEMG data (sEMG area) and frequency-domain parameters (MNF and MDF). Thus, the null hypothesis that the data are normally distributed was not rejected, and we considered it appropriate to apply parametric statistics. Moreover, considering the small sample, we paid particular attention to ensure that the Levene’s test provided values of *p* greater than 0.05 for each parametric comparison.

The comparisons of sEMG area, MNF and MDF, between the two groups for each single muscle, were performed using Student’s *t*-test. Two-way analysis of variance (ANOVA) for repeated measures was used to detected significant differences between the two frequency-domain features (MNF and MDF); the variation between the two parameters for each muscle was the “within” repeated factor (frequency parameters) and the variation between the two groups as “between” factor (group). The interaction between the two factors was also computed. One-way ANOVA with repeated measures was performed to detect changes in sEMG amplitude and frequency parameters over the cycles of movement, in each group. In this case, the F-statistic was adjusted applying Greenhouse–Geisser correction, which produces a more conservative *p*-value. For all the ANOVA procedures, the magnitude of the effect was evaluated by partial eta-squared (*η*^2^*_p_*). 

To further characterize the sEMG power spectrum, the profiles of PSD patients and healthy participants were analyzed by means of the principal component analysis (PCA). The PCA is a technique for reducing a large number of variables describing a data set to a smaller number of independent factors or principal components (PCs). That is, the first principal component (PC1) symbolizes the most common profile across the profiles of the PSD, and the second principal component (PC2) is the next most common, after the PC1 is accounted for. To simplify the interpretation of PCs, we applied varimax rotation procedure in which the PCs axes are rotated and the correlations in the rotated component matrix are close to 1, −1 or 0. Associated to the extraction of PCs, we calculated the weighting coefficients. These indices represent the Pearson correlation coefficients between the elements of the original dataset and each of the PCs, assuming any value in the interval between −1 and 1. This procedure allows to quantify the contribution of the original PSD profiles to each PC, that is, each PC can be associated with patients or healthy individuals. 

The correlation between changes in each of the two frequency-domain features and the level of disease severity was determined by computing the Pearson correlation coefficients (*r*) and the related level of statistical significance (*p*). Disease severity was assessed by FEV1 and FEV1/FVC tests.

For all the statistical tests, the level α was established at 0.05. Statistical analysis was performed by using SPSS version 27 (SPSS, Inc., Chicago, IL, USA, IBM, Somers, NY, USA).

## 3. Results

Almost all participants performed the entire 10 cycles of movement. However, some participants anticipated the last cycle making the final flexion–extension movement irregular. Thus, in order to have consistent statistics across subjects, we performed the analyses over nine cycles.

### 3.1. Changes in EMG Amplitude and Frequency-Domain Parameters

**Figure 2:**
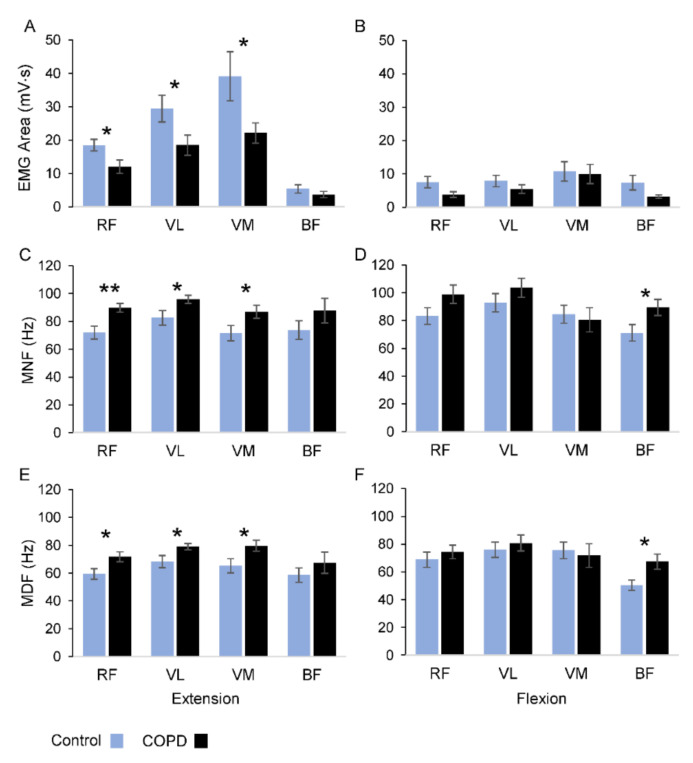
Summary of statistical results of electromyographic parameters. Comparison of sEMG amplitude (**A**,**B**), MNF (**C**,**D**) and MDF (**E**,**F**) between COPD patients (black) and healthy individuals (azure) for each muscle. In the left panel are the values measured during extension movements, while in right panel are the values measured during flexion movements. Abbreviations: MNF = mean frequency; MDF = median frequency; RF = Rectus Femoris; VL = Vastus Lateralis; VM = Vastus Medialis; BF = Biceps Femoris. The data are expressed as means and standard errors; * *p* < 0.05; ** *p* < 0.01.

During the extension movements most of the sEMG activity occurred in RF, VL and VM muscles (Figure 2A), with the group of patients showing significantly lower levels of sEMG area with respect to the control group (RF: *p* = 0.026; VL: *p* = 0.043; VM: *p* = 0.047). No significant changes were observed over the four muscles for the flexion movements (Figure 2B). Both groups showed similar levels of sEMG activity over the movement cycles, with no significant differences for all the muscles, except for the VL muscle in the control group (*F*_8_ = 3.227, *p* = 0.036, *η*^2^*_p_* = 0.26).

Changes in frequency-domain parameters are illustrated in Figure 2C–F, and the statistical analysis is summarized in Table 3. The two frequency parameters showed statistically significant differences, in both extension and flexion movements, for all the muscles, with the MNF exhibiting higher values than MDF (see columns of frequency parameters in Table 3 and compare Figure 2C,D with Figure 2E,F).

Statistical differences between groups were detected for the RF, VL and VM muscles during the extension movements and for the BF muscle during flexion movements (see group columns in Table 3). The average values of MNF for the extension movements were higher in the COPD group with respect to the control participants (Figure 2C). Applying local *t*-tests, significant changes in MNF values between the groups were detected for RF (+25%; *p* < 0.01), VL (+16%; *p* = 0.041) and VM (+22%; *p* = 0.045) muscles, but not for BF (*p* = 0.224). During the flexion movements (Figure 2D), significant changes in MNF values were observed only for the BF muscle (+26%; *p* = 0.041). The pattern of variations observed for MNF was replicated for MDF during both movement directions (Figure 2E,F). In fact, during the extensions, the MDF values were significantly higher in COPD patients than in the control group for RF (+21%; *p* = 0.029), VL (+16%; *p* = 0.041) and VM (+22%; *p* = 0.039), but not for BF (*p* = 0.342) muscles. Changes in MDF over the flexion movements were detected only for the BF muscle (+34%; *p* = 0.019).

No interaction between group and frequency parameters was observed, except for a marginal, significant interaction observed in the RF muscle during extension movements (*p* = 0.06). The values of the MDF and MNF showed no significant changes over the movement cycles for all the muscles in both groups, except for the VM in the control group, regarding the MDF measure (*F*_8_ = 3.405, *p* = 0.02, *η*^2^*_p_* = 0.27).

**Figure 3:**
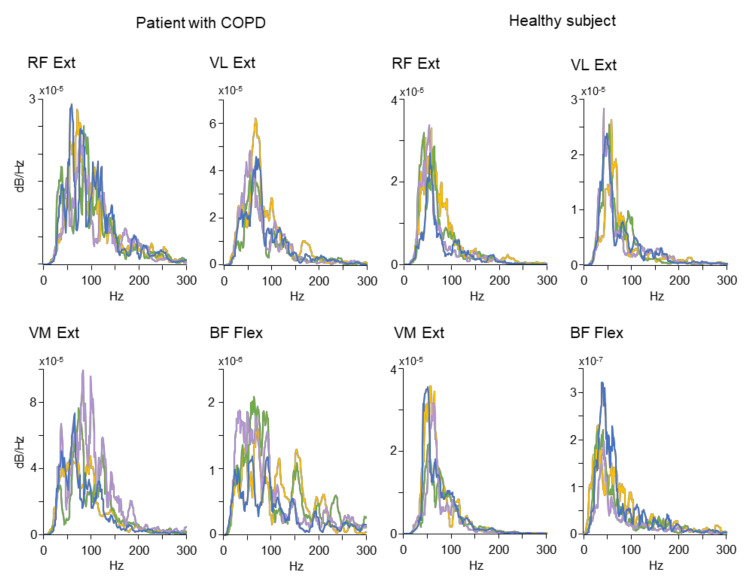
Representative examples of Power Spectrum Density of sEMG recorded in a patient with COPD (the four panels on the **left**) and in a healthy subject (the four panels on the **right**). Each panel contains four spectral profiles obtained from the second (blue line), the fourth (purple line), the sixth (green line) and the eighth (yellow line) leg extension (Ext) or flexion (Flex) movements. Power spectra regarding the RF, VL and VM were obtained during the extension movements, while the spectrum regarding the BF was obtained during flexion movements. For other abbreviations see Figure 2.

A sample of original PSD distributions used to compute the MNF and MDF is depicted in Figure 3. The plots represent spectral profiles obtained from a patient with COPD (the four plots on the left) and a healthy participant (the four plots on the right). For the sake of clarity, each plot includes PSD profiles from four out of nine leg movements. Only the data associated with the statistically significant results reported for the MNF and MDF (see Figure 2) were included in Figure 3. Specific patterns of spectrum power profiles emerge from the plots: the healthy subject tended to exhibit peaked profiles mostly localized in the left side of the spectrum, while the COPD patient showed profiles with larger frequency bandwidth shifted toward the portion of the spectrum characterized by higher frequencies. 

### 3.2. Principal Component Analysis of PSD Profiles

**Figure 4:**
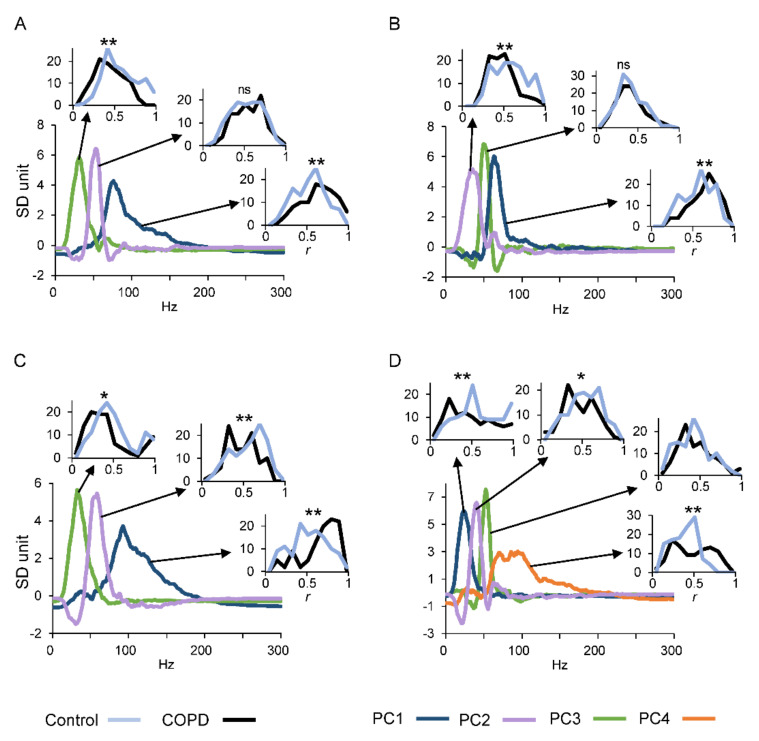
Principal component analysis derived from power spectral profiles. We reported the first three principal components for RF, VL and VM during extension movements (**A**–**C**) and four principal components for BF during flexion movements (**D**). The inserts show distribution histograms of the weighting coefficients obtained from the correlations between each PC of each muscle and single frequency profile recorded in COPD (black) and healthy (azure) groups. Abbreviations: PC1–PC4 = first to fourth principal component; *r* = coefficient of correlation; * *p* < 0.05; ** *p* < 0.01. For other abbreviations and symbols see Figure 2.

To obtain a statistical summary of the frequency profiles observed over the dataset including all the participants, and to identify the most common patterns for each of the two groups, we performed the PCA. In Figure 4A–D and in Table 4 are summarized the results of this analysis. The largest plots inserted in each panel of Figure 4 illustrate the PCs explaining most of the total variance present in the original dataset (see Table 4) for each muscle. Thus, these PCs represent the typical spectral profiles for extensor muscles during the extension movements (Figure 4A–C) and for the flexor muscle during the flexion movements (Figure 4D). The small plots inserted in the panels illustrate the distribution of the level of correlation, expressed as coefficient of correlation (*r*), between each PC and each original spectral profile from the common dataset of the two groups of participants. 

In the case of the RF muscle (Figure 4A and Table 4), the first PC (blue line) explains 32% of the total variance and covers a large portion of the spectrum characterized by higher frequencies. The distribution of the correlations between the first PC and each profile within the dataset of the group of patients (black line, *r* mean value ± SD: 0.58 ± 0.22) was significantly greater (*p* < 0.001) than the coefficients obtained for the control participants (azure line, *r* mean value ± SD: 0.47 ± 0.19). 

An opposite behavior was observed for the third PC (green line, 25% of the total variance), which had the profile shifted towards the low frequencies, and the control group showed a better correlation with respect the COPD patients (control *r* mean value ± SD: 0.52 ± 0.2; COPD *r* mean value ± SD: 0.38 ± 0.18; *p* < 0.001).

The second PC (lilac line) explained 26% of the total variance and covered an intermediate position over the frequency distribution. In this case, no significant differences were observed between the two groups regarding the distribution of the correlation coefficients.

A similar pattern was observed for the other muscles (Figure 4B–D). In fact, when the PCs represent the spectral profiles shifted toward the higher frequencies (blue lines for VL and VM and orange line for BF), the correlations with the observed data were higher in the patients with COPD than in control individuals. On the contrary, PCs representing spectral profiles shifted toward the lower frequencies (lilac line for VL, green line for VM and blue line for BF) were better correlated with the dataset of control group than the dataset of the COPD group. We did not consider the higher-order PCs, as each of them explained a very small fraction of the variance hardly distinguishable from noise. Noteworthy, in the cases of spectral profiles associated with the COPD group, they not only were shifted to the higher frequencies, but their shapes were asymmetric, with a wider tail towards the high frequencies.

Detailed numerical data for all muscles and movements are reported in Table 4. By inspection of this table, it is possible to observe that when the muscles acted as antagonist, no homogeneous pattern was detected, with a reduced number of cases showing significant differences for the correlation coefficients between the two groups.

### 3.3. Regression Analysis

In Table 5 are summarized the results of linear regression analysis performed to correlate the two frequency-domain parameters with the level of disease severity, evaluated by means of FEV1 and FEV1/FVC. The correlations with statistical significance (*p* < 0.05) occurred only for extension movements, with MDF and MNF exhibiting good or strong levels of correlation with respect to the disease severity for almost all the extensor muscles.

## 4. Discussion

The results of the present study show that a non-fatiguing motor task is effective for identifying and quantifying the increase in frequency range of the sEMG power spectrum, along with the concomitant decrease in sEMG magnitude, in persons with COPD compared to a group of healthy individuals. Noteworthy, the changes in both MNF and MDF of the sEMG signals were correlated with disease severity (i.e., the greater the frequency, the greater the disease severity), thus further supporting the use of this functional task within a clinical setting to classify the patients based on their impairment in muscle function.

The decreased magnitude of the sEMG signals in persons with COPD is consistent with other studies [19,25] and should be associated with the disease-induced muscle dysfunctions, such as reduced muscle size, muscle weakness and changes in fiber composition. The level of sEMG amplitude was stable over the movements, indicating the non-fatiguing nature of the motor task adopted in the current study.

To the best of the authors’ knowledge, this is the first study reporting increased frequency of the sEMG spectrum (i.e., MFD and MNF) during a non-fatiguing movement task in patients with COPD. In previous studies, the changes in the sEMG power spectrum were used to quantify the level of fatigue associated with COPD during incremental exercise tests leading to participant exhaustion [7,19,20,21,24]. As force-incremental motor tasks are suitable to evoke muscle fatigue, the estimation of fiber composition based on frequency-domain features could be uncertain. In fact, when fatigue procedures are used at high strength levels, the force generated does not depend on the fiber composition, but on the increase in motor unit firing [23]. Moreover, exercises with increased energy demand may create discomfort in patients with COPD, thus further reducing muscle oxygenation and aerobic metabolism, with a tendency to recruit more type II than type I muscle fibers. The effects of fatiguing motor tasks on the muscle fiber recruitment could be explained by the variability showed by MDF and MNF when these frequency-domain parameters have been used at a high muscle contraction level. The MDF is less affected by random noise, but more affected by muscle fatigue [28]. On the same line, increasing temperature during incremental contraction affects the value of the MNF [29]. In fact, the MNF was found to be more sensitive in differentiating patients with COPD from control subjects at lower than higher levels of force expression [7,24].

In the current study, the increase in MDF and MNF in patients with COPD compared with healthy people, and the stability showed by the two frequency-domain parameters over the leg movements, indicate that the limitations in the ability of sEMG analysis to reliably detect the changes in muscle fiber distribution could be attenuated by adopting non-fatiguing leg flexion–extension movements. Furthermore, the PCA performed in the current study provided further insight into the characteristics of power profiles associated with COPD, allowing to quantify the most common profile shapes of the power frequency spectrum within each experimental group. This made it possible to evaluate any visible differences in frequency bandwidth over the power spectrum, and to spot any potential skewing in the shape of the profiles. In fact, not only were the mean and median values of the frequency spectrum higher in COPD compared to healthy participants, but also the most common spectral profiles in COPD patients were skewed to the right, indicating a peculiar change in the shape of the patient power spectrum, with a longer tail towards the higher frequencies. 

Another relevant result of this study regards the good correlations between each of the two frequency paraments and the level of disease severity. This point strengthens the possibility that the shift of power spectrum toward higher frequencies observed in patients with COPD is associated with switching from type I to type II fibers observed by means of invasive techniques. In fact, the MDF and MNF changes correlated with changes in the FEV1 and FEV1/FVC, showing negative relations and levels of correlation comparable with the correlation between the same marker of disease severity and the proportions of type II fibers measured by histological and molecular techniques in several studies reported in the meta-analyses performed by Gosker et al. [11].

The results of the present study support the adoption of a non-fatiguing task as a valuable and easy-to-administer tool to better estimate muscle fiber composition by identifying and quantifying the disease-induced increase in sEMG frequency spectrum. Due to the non-fatiguing nature of this task, the patients were not asked to experience any strenuous effort that may create discomfort, especially for those patients with moderate to severe levels of disease.

A non-invasive monitoring of the muscle fiber composition could be of great utility not only in the characterizing the level of disease severity, but also to monitor the effects of rehabilitation protocols addressed to restore skeletal and ventilatory muscle functionality. In fact, neuroplasticity of motor units has been well documented both in animals and humans [30]; for example, a significant shift of motor units can be guided by the type of muscular activity [31]. Therefore, it is possible that a specific physical training can restore, at least in part, the original composition of muscle fibers and improve muscle function in COPD patients. On this line, Ovechkin et al. [22] showed that respiratory muscle training induced improvements in respiratory muscles, modulating the activation of slow and fast twitch fibers and reversing maladaptive neuromuscular changes observed in patients with COPD. These authors used the sEMG power spectrum to monitor the changes in activity of type I and II fibers before and after training of several respiratory muscles. Similar neuromuscular transformations were found in peripheral muscles by using needle biopsies of the right vastus lateralis muscle before and after rehabilitation training. In particular, a decrease in type IIx muscle fiber proportion and an increase in type I muscle fiber proportion have been reported in COPD after cycling endurance or interval training [32,33].

The results of these studies highlight the need to employ a test to characterize muscle fiber composition and the associated adaptations after rehabilitation training. The results of the current work provide an experimental support in considering the power spectrum analysis of sEMG, obtained during a simple and affordable task for patients with COPD, as a valuable tool to assess and possibly validate the training adaptive changes in muscle fiber composition having clinical significance. 

It is noteworthy that in our task the magnitude of muscle activation was remarkably greater during extension than flexion phases of the movement. In fact, the knee extensor muscles (i.e., RF, VM and VL) were activated to counteract the effects of gravity in the extension phase of the movement. On the contrary, the flexion phase occurred along the line of gravity, and the magnitude of muscle activation was marginal (see Figure 2B) due to a relevant movement control carried by joint passive viscoelastic resistance [34,35,36,37,38]. This task-dependent condition may also explain the more consistent pattern of differences observed between groups during extension than flexion movements for MDF and MNF (see Figure 2C–F) and for PCA (see Table 4). In general, it could be important to consider the biomechanical condition of the motor task to which the sEMG power spectrum analysis is applied, in particular for natural tasks, such as cycling and walking. In fact, in these tasks each muscle alternates the role of agonist and antagonist, and the contribution of a passive component can have significant variations. Adopting a simple task, such as the one used in this study, can help to distinguish the role for each muscle and to interpret the outcome of sEMG power analysis correctly.

## 5. Limitations

A limited sample size was used for this study, which might weaken the outcome and robustness. However, we tried to compensate for this limit by providing different statistical control tests, such as the Shapiro–Wilk test to check for normality and Levene’s test to check for homogeneity of the variance between the groups. These tests showed acceptable levels of significance. Moreover, the good level of significant differences between the two groups (*p* values) and the magnitude of the experimental effect (*η*^2^*_p_*) make us confident that the data reported in the current study can also be confirmed with a larger sample. 

The experimental design did not include histological or cellular examinations to achieve a direct determination of the muscle fiber composition. These data would have provided a greater strength to the correlations between changes in sEMG parameters and changes in fiber type distribution. The correlation of our sEMG data with the disease severity and the large use of the quadriceps muscle in many studies examining peripheral muscle biopsies of patients with COPD [1,6,8,9,10,11,18] lead us to predict that this correlation may be demonstrated in future studies.

## 6. Conclusions

The changes in frequency-domain features in patients with COPD and the good correlation with the disease severity, reported in the current paper, suggest that sEMG power spectrum analysis applied to a non-fatigable motor task may be suitable for estimating muscle fiber composition. Moreover, the non-invasive nature of this approach encourages to use this paradigm to monitor the progression of disease severity and the effects of physical rehabilitation programs.

## Figures and Tables

**Table 1:** Anthropometric data for the patients with COPD and healthy individuals.

	Patients				Healthy			
#	Gender	Age (y)	Weight (kg)	Height (cm)	Gender	Age (y)	Weight (kg)	Height (cm)
1	F	73	64	145	M	65	63	159
2	M	50	87	183	M	60	85	179
3	M	74	60	170	M	67	90	174
4	F	39	52	156	F	63	65	163
5	F	80	67	165	M	78	68	169
6	M	68	51	171	F	68	70	163
7	F	64	79	167	M	61	65	165
8	M	56	80	168	F	63	62	163
9	F	60	58	157	F	58	84	153
10	M	66	90	166	F	58	49	152

Abbreviations: F = female; M = male.

**Table 2:** Clinical data for the patients with COPD.

#	Smoker	Years of Smoking	GOLD Group	FEV1	FVC	FEV1/FVC	D_LCO_	mMRC
1	NO	/	C	113%	114%	67%	97%	1
2	EX	50	A	64%	100%	41%	105%	0
3	YES	50	A	75%	62%	63%	122%	1
4	NO	/	D	39%	56%	54%	97%	2
5	EX	40	C	75%	113%	47%	94%	1
6	EX	40	B	27%	86%	21%	35%	2
7	EX	40	B	70%	91%	56%	86%	2
8	EX	30	D	33%	47%	52%	55%	4
9	NO	/	D	77%	85%	67%	73%	1
10	EX	20	C	69%	99%	47%	89%	2

Abbreviations: EX = before; GOLD = Global Initiative for Chronic Obstructive Lung Disease; FEV1 = forced expiratory volume in one second; FVC = forced vital capacity; FEV1/FVC = ratio between two terms; D_LCO_ = diffusing capacity for carbon monoxide; mMRC = modified Medical Research Council Dyspnea scale.

**Table 3:** Summary of the two-way ANOVA comparing groups and frequency parameters.

		Extension			Flexion		
		Group	Frequency Parameters	Group vs. Frequency Parameters	Group	Frequency Parameters	Group vs. Frequency Parameters
	*F*	8.385	123.165	4.026	1.901	53.717	3.580
RF	*p*	**0.010**	**<0.001**	0.060	0.185	**<0.001**	0.075
	*η* ^2^ *_p_*	0.32	0.87	0.18	0.10	0.75	0.17
	*F*	5.060	140.310	0.881	0.869	66.215	1.493
VL	*p*	**0.037**	**<0.001**	0.360	0.364	**<0.001**	0.238
	*η* ^2^ *_p_*	0.22	0.89	0.05	0.05	0.79	0.08
	*F*	5.051	17.072	0.106	0.131	119.704	0.020
VM	*p*	**0.037**	**<0.001**	0.748	0.722	**<0.001**	0.889
	*η* ^2^ *_p_*	0.22	0.49	0.01	0.01	0.87	0.01
	*F*	1.294	139.996	2.645	5.929	113.413	0.097
BF	*p*	0.270	**<0.001**	0.121	**0.026**	**<0.001**	0.759
	*η* ^2^ *_p_*	0.07	0.89	0.13	0.25	0.86	0.01

Abbreviation: RF = Rectus Femoris; VL = Vastus Lateralis; VM = Vastus Medialis; BF = Biceps Femoris; *F* = critical value for statistical significance; *p* = probability value for statistical significance; *η*^2^*_p_* = partial eta-squared; in bold the statistically significant differences.

**Table 4:** Summary of Principal Component Analysis applied to power spectrum profiles.

		Extension				Flexion			
		PC1	PC2	PC3	PC4	PC1	PC2	PC3	PC4
RF	Proportion	32%	26%	25%		27%	20%	16%	
	COPD (*r*)	0.58	0.49	0.38		0.51	0.38	0.34	
	Control (*r*)	0.47	0.46	0.52		0.43	0.4	0.36	
	*p*	**<0.001**	0.223	**<0.001**		**0.012**	0.594	0.391	
VL	Proportion	34%	26%	15%		25%	18%	16%	15%
	COPD (*r*)	0.59	0.41	0.34		0.44	0.36	0.38	0.37
	Control (*r*)	0.51	0.51	0.34		0.44	0.39	0.34	0.3
	*p*	**0.007**	**<0.001**	0.99		0.987	0.294	0.172	**0.008**
VM	Proportion	34%	26%	23%		31%	24%		
	COPD (*r*)	0.62	0.42	0.34		0.42	0.47		
	Control (*r*)	0.47	0.52	0.44		0.55	0.33		
	*p*	**<0.001**	**<0.001**	**0.01**		**<0.001**	**<0.001**		
BF	Proportion	34%	28%	18%	11%	30%	23%	17%	17%
	COPD (*r*)	0.5	0.46	0.33	0.25	0.42	0.39	0.38	0.41
	Control (*r*)	0.53	0.42	0.41	0.28	0.52	0.47	0.35	0.31
	*p*	0.396	0.312	**0.004**	0.149	**0.006**	**0.012**	0.328	**<0.001**

Abbreviations: PC1–PC4 = first to fourth principal component; Proportion indicates the fraction of the total variance accounted by each PC; *r* = coefficient of correlation. In bold the statistically significant differences. For other abbreviations see Table 3.

**Table 5:** Simple linear correlation between frequency parameters and disease severity.

		Extension				Flexion			
		FEV1		FEV1/FVC		FEV1		FEV1/FVC	
		*r*	*p*	*r*	*p*	*r*	*p*	*r*	*p*
MNF	RF	**−0.85**	**0.002**	**−0.66**	**0.038**	−0.05	0.881	−0.20	0.585
	VL	−0.60	0.069	**−0.79**	**0.006**	0.24	0.496	−0.10	0.779
	VM	**−0.71**	**0.022**	**−0.72**	**0.019**	0.35	0.321	0.00	0.993
	BF	−0.03	0.944	−0.03	0.942	−0.06	0.858	−0.41	0.238
MDF	RF	**−0.90**	**0.001**	**−0.79**	**0.007**	−0.14	0.705	−0.22	0.545
	VL	**−0.70**	**0.024**	**−0.74**	**0.015**	0.37	0.286	−0.01	0.968
	VM	**−0.83**	**0.003**	−0.58	0.076	0.37	0.300	0.03	0.941
	BF	−0.06	0.866	−0.05	0.883	−0.08	0.829	−0.40	0.255

Abbreviations: MNF = Mean Frequency; MDF = Median Frequency; FEV1 = score of forced expiratory volume in 1 s; FVC = score of forced vital capacity FEV1/FVC = the ratio of FEV1 to FVC. In bold the statistically significant correlations. For other abbreviations see Table 3 and Table 4.

## Data Availability

The data that support the findings of this study are available on requestfrom the corresponding author, M.S.V. The data are not publicly available due to containing information that could compromise the privacy of research participants.
